# A8 GREATER DISEASE BURDEN AMONG CANADIANS WITH CELIAC DISEASE MOST ECONOMICALLY DEPRIVED: FINDINGS FROM THE NATIONWIDE STATE OF CELIAC SURVEY

**DOI:** 10.1093/jcag/gwae059.008

**Published:** 2025-02-10

**Authors:** J A King, C McAuley, M Secord, M pinto-sanchez, J Turner, D Gidrewicz, S Case, D Duerksen

**Affiliations:** Community Health Sciences, University of Calgary, Calgary, AB, Canada; Canadian Celiac Association, Mississauga, ON, Canada; Canadian Celiac Association, Mississauga, ON, Canada; McMaster University, Hamilton, ON, Canada; University of Alberta, Edmonton, AB, Canada; Community Health Sciences, University of Calgary, Calgary, AB, Canada; Canadian Celiac Association, Mississauga, ON, Canada; University of Manitoba, Winnipeg, MB, Canada

## Abstract

**Background:**

Individuals with celiac disease (CeD) most economically deprived may face challenges adhering to the gluten-free diet (GFD). However, it is less clear how income may be associated with symptoms or comorbidity.

**Aims:**

To determine symptom and comorbidity status across the economic spectrum among Canadians living with CeD.

**Methods:**

At the end of 2022, the State of Celiac Disease in Canada Survey was administered to individuals living with CeD in Canada. Questions on GFD adherence, symptoms, and comorbidities were recorded by respondents. Participants who did not provide information on their household income (before tax) were excluded from analysis. The total number of symptoms and comorbidities were calculated per respondent, with symptoms further stratified into gastrointestinal (*n*=10), non-gastrointestinal (*n*=15), and neuropsychiatric (*n*=5). Tests of proportions and Kruskal-Wallis tests were used to assess for differences in outcomes across income levels. The Benjamini-Hochberg correction was used to account for multiple testing.

**Results:**

A total of 3,950 respondents were included. Significant differences in gastrointestinal symptoms before and after diagnosis were detected across income groups (e.g., *p*<0.05 for those under $30,000 compared to all other income groups). Similar findings were present for non-gastrointestinal symptoms (e.g., *p*<0.01 for those under $30,000 compared to all other income groups except $30,000–49,999 before diagnosis) and neuropsychiatric symptoms (e.g., *p*<0.01 for those under $30,000 compared to all other income groups). The number of comorbidities also differed significantly across certain income levels (e.g., *p*<0.01 for those under $30,000 compared to all other income groups). There were no differences in accidental gluten consumption across income categories, although a borderline significant difference (*p*=0.06) was observed for intentional gluten consumption between those with income under $30,000 compared to those with income greater than $150,000.

**Conclusions:**

There are notable differences in CeD across an economic gradient, with those in lower income brackets living with a greater number of symptoms and comorbidities. Further attention is warranted to minimize additional burden from CeD among individuals with limited income.

Comorbidity status and gluten consumption across income levels

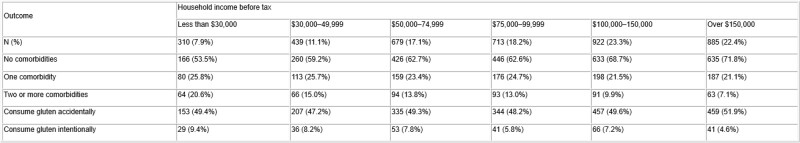

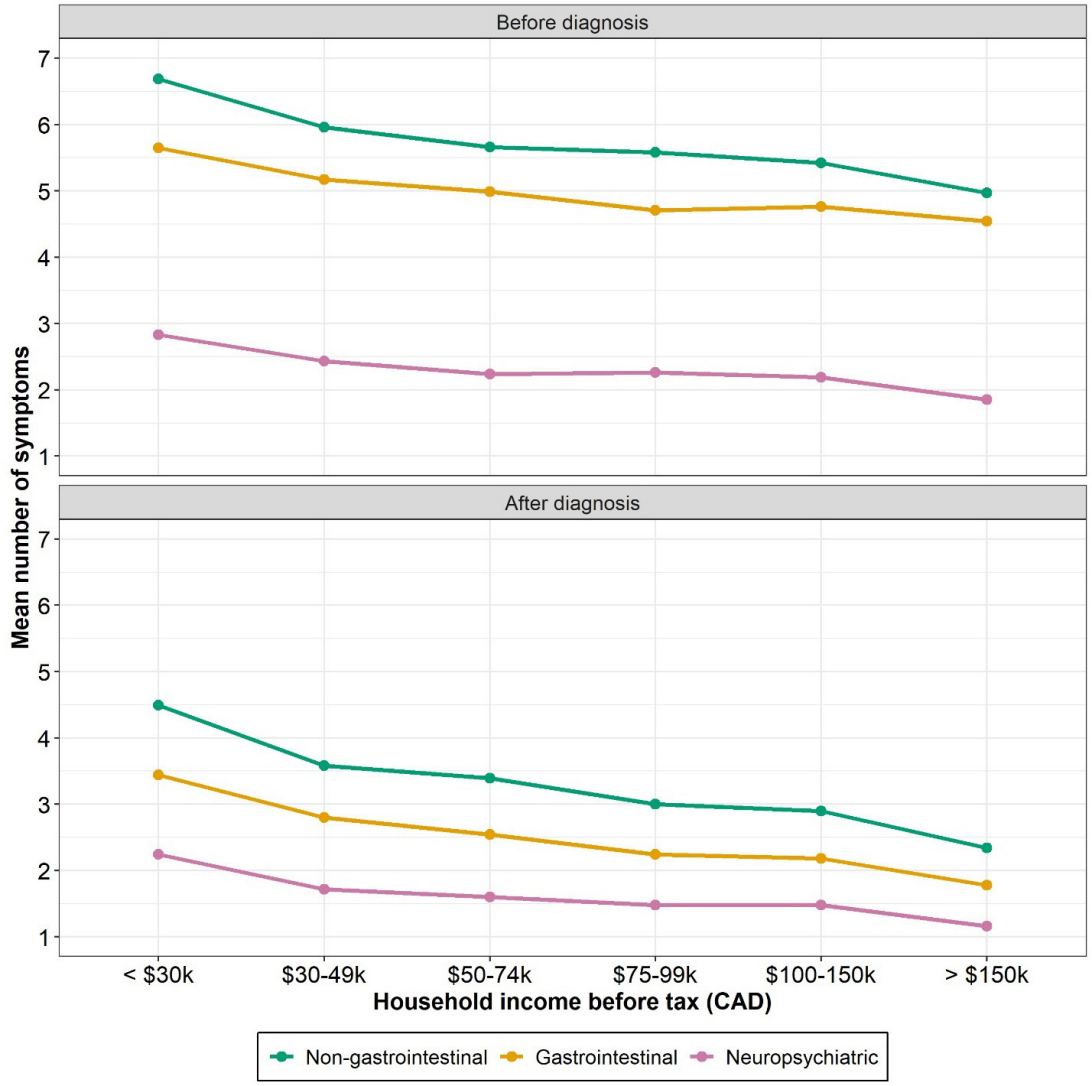

Symptoms experienced before and after diagnosis across income level

**Funding Agencies:**

Celiac Canada

